# The Overexpression of *Acyl-CoA Medium-Chain Synthetase-3 (ACSM3)* Suppresses the Ovarian Cancer Progression *via* the Inhibition of Integrin β1/AKT Signaling Pathway

**DOI:** 10.3389/fonc.2021.644840

**Published:** 2021-03-31

**Authors:** Limei Yan, Zeping He, Wei Li, Ning Liu, Song Gao

**Affiliations:** Department of Obstetrics and Gynecology, Shengjing Hospital of China Medical University, Shenyang, China

**Keywords:** ovarian cancer, acyl-CoA medium-chain synthetase-3, integrin β1, proliferation, metastasis

## Abstract

Ovarian cancer is considered as one of the most fatal gynecologic malignancies. This work aimed to explore the effects and regulatory mechanism of *Acyl-CoA medium-chain synthetase-3* (*ACSM3*, a subunit of CoA ligases) in ovarian cancer progression. As well as employing CCK-8 assay, clone formation assay, and cell cycle analysis were carried out to investigate cell proliferation ability. Wound healing assay and transwell assay were subsequently used to assess cell migration and invasion. Mice xenografts were then conducted to measure the effects of *ACSM3* on tumor development *in vivo*. Our bioinformatics analysis suggested that the expression of *ACSM3* was down-regulated in ovarian cancer tissues, and the low expression level of *ACSM3* might related with poorer overall survival than high mRNA expression of *ACSM3* in ovarian cancer patients. We artificially regulated the expression of *ACSM3* to evaluate its effects on ovarian cancer malignant phenotypes. Our data revealed that the overexpression of *ACSM3* inhibited cell proliferation, migration, and invasion of ovarian cancer cells. In contrast, the knock-down of *ACSM3* received the opposite results. Our western blot results showed that the Integrin β1/AKT signaling pathway was negatively regulated by *ACSM3* expression. Moreover, *ACSM3* overexpression-induced suppression of cell migration and invasion activities were abolished by the overexpression of *ITG β1* (Integrin β1). Additionally, the growth of ovarian cancer xenograft tumors was also repressed by the overexpression of *ACSM3*. And *ACSM3* interference obtained the contrary effects *in vivo*. In summary, *ACSM3* acts as a tumor suppressor gene and may be a potential therapeutic target of ovarian cancer.

## Introduction

Ovarian cancer is considered one of the most fatal gynecologic malignancies (1). Due to the asymptomatic development, ovarian cancer is frequently not diagnosed until at an advanced and incurable stage, which is seen as a silent killer (2). When ovarian cancer is diagnosed at an early stage that grows in one or two sides of ovaries, the cure rate of it could reach 90% (3). However, the cure rate of ovarian cancer attenuates substantially with the metastasis of the tumors to the uterus and bladder (stage II), the peritoneal cavity (stage III), or the visceral organs (stage IV) (3). More than 70% of patients with ovarian cancer are not diagnosed until tumors are actively metastasized and developed to stage III or IV (4). Unlike hematogenous disseminating cancers, ovarian cancer metastasizes throughout the peritoneal cavity by peritoneal fluid instead of vasculature (5). Then, ovarian cancer tumors compress the visceral organs and even spread to the parenchyma of the lung or liver (5). The metastasis and chemo-resistance cause the death of ovarian cancer patients (6). Despite abundant research on the pathogenesis and therapy for ovarian cancers, there is still a lack of authoritative treatment. Therefore, it is essential to find new potential target for effectual therapy in ovarian cancer.

*Acyl-CoA medium-chain synthetase-3* (*ACSM3*) is a subunit of CoA ligases that plays a significant role in the progression of many diseases. Ruan et al. (7) reported that *ACSM3* had a down-regulated expression in hepatocellular carcinoma, which enhanced the metastasis *via* the AKT-WKN1 signaling pathway. Zhu et al. (8) confirmed that the loss of *ACSM3* was associated with poor prognosis and immune exclusion in malignant melanoma. Peter et al. (9) reported that the expression of *ACSM3* was decreased in ulcerative colitis and might be related to butyrate oxidation. However, the effects of *ACSM3* on ovarian cancer has not been reported.

Integrin β1 (*ITG β1*), a member of the heterodimeric transmembrane receptors family, is known as a cell-extracellular matrix (ECM) adhesion protein (10). Integrin β1 exerts regulatory functions on cellular processes including cell proliferation, migration, and invasion *via* the signals reacted to ECM during cancer progression (10). Studies indicated that *ITG β1* (Integrin β1) had an up-regulation in ovarian cancer tissues and the overexpression of *ITG β1* (Integrin β1) was linked with poor prognosis as well as higher clinical stages (11, 12). The up-regulated expression of *ITG β1* (Integrin β1) elevates the abilities of cell migration and invasion in ovarian cancer tumor (11).

In this work, we found that the expression of *ACSM3* was down-regulated in ovarian cancer by bioinformatics analysis, and the patients with low expression of *ACSM3* showed poor overall survival. We artificially regulated the expression of *ACSM3* to evaluate the effects on ovarian cancer progression. The modulation of *ACSM3* markedly influenced cell proliferation, metastasis, and invasion *in vitro* as well as the growth of ovarian cancer tumors *in vivo*. Further work showed that these effects are associated with its regulation on the Integrin β1/AKT signaling pathway.

## Materials and Methods

### Cell Culture, Vectors Construction, and Cell Transfection

OV-90, SK-OV-3, OVCAR-3, and A2780 cells were purchased from Procell Life Science&Technology Co., Ltd (Wuhan, China). OV-90 and OVCAR-3 cells were maintained in a specific medium (Procell). SK-OV-3 cells were cultured in McCoy’s 5A medium (Procell). A2780 cells were cultured in DEME (Gibco Life Technologies, NY, USA). All the medium was supplemented with 10% fetal bovine serum (FBS). And cells were incubated in 5% CO_2_ at 37°C.

Lentiviral vectors expressing small hairpin RNA (shRNA) targeting *ACSM3* were named Lv-shRNA1-ACSM3 or Lv-shRNA2-ACSM3. The complementary cDNAs of *ACSM3* were synthesized and the lentiviral overexpressed vectors pcDNA3.1 (GenScript, Nanjing, China) of *ACSM3* were constructed as Lv-ACSM3. The shRNA sequences were listed as follow. shRNA1-ACSM3: CGATGTTAAGATTGTAGATGT. shRNA2-ACSM3: GCTTGTACAGAATGATATAAC. The Lv-shRNA1-ACSM3 or the Lv-shRNA2-ACSM3 were infected into OV-90 cells. The Lv-ACSM3 was infected into A2780 or SK-OV-3 cells. The overexpressed *ITG β1* (Integrin β1) vectors were transfected into A2780 cells by Lipofectamine 3000 (Invitrogen, Carlsbad, California, USA) according to the manufacturer’s instructions.

### Western Blot

Total protein was extracted from infected cells or tumors, respectively. The protein concentration was measured by BCA Protein Assay Kit (Solarbio, Shanghai, China). 20 μL protein samples were resolved by 12% SDS-PAGE and transferred onto PVDF membranes (Millipore, Billerica, MA, USA). Protein bands were incubated with primary antibodies at 4°C overnight after being blocked with 5% skim milk. Then, membranes were incubated with HRP labeled secondary antibodies (Solarbio; 1:3000) at 37°C for an hour. Protein probes were imaged with an ECL reagent (Solarbio). The primary antibodies were ACSM3 (Proteintech. Wuhan, China; 1:500), Integrin β1 (Proteintech; 1:500), E-cadherin (ABclonal, Shanghai, China; 1:1000), cyclin D1 (ABclonal; 1:500), p-AKT (ABclonal; 1:500), AKT (ABclonal; 1:1000), Vimentin (ABclonal; 1:500), c-Myc (ABclonal; 1:500), and GAPDH (Proteintech; 1:10000).

### Cell Counting Kit-8 Assay (CCK-8 assay)

The ability of cell proliferation was detected by CCK-8 assay. Infected cells (4×10^3^ per well) were plated into 96-well plates and were cultured for 0 h, 24 h, 48 h, 72 h, and 96 h. Then, a 10 μL CCK-8 kit (Keygen Biotech, Jiangsu, China) was added to each well and incubated for two hours. The OD value was measured at 450 nm.

### Cell Cycle Analysis

Nuclear DNA content was evaluated by fluorescence-activated cell sorting. Infected cells were deprived of FBS and were rinsed with phosphate buffer saline (PBS) three times. Cells were maintained in pre-cooling 70% ethanol at 4°C for 2 h and rinsed with PBS. After being resuspended, cells were incubated with propidium iodide (50 μL/mL; Beyotime, Shanghai, China) and RNAase (0.1 mg/mL; Beyotime, Shanghai, China) at 37°C for half an hour protecting from the light. The nuclear DNA content was measured by performing a NovoCyte flow cytometer (ACEA Biosciences, Hangzhou, China).

### Wound Healing Assay

Equal numbers of OV-90, A2780, and SK-OV-3 cells were plated into 6-well plates after being infected for 24 h, respectively. A wound was achieved by 200 μL pipette tips in each well after cells were cultured for 24 h. Cells were rinsed by the medium without serum. The distance between cells was photographed at 0 h or 24 h, respectively.

### Transwell Assay

After being infected for 24 h, cells were collected and applied to top chambers of transwell inserts (Corning Incorporated, NY, USA) with a non-serum medium. 900 μL medium with 10% FBS was added into lower chambers and the systems were maintained for 24 h. Cells were further stained with 0.5% crystal violet (Amresco, Shanghai, China). The number of migrated cells in lower chambers was quantified and revealing the invasion ability of cells.

### Tumor Xenograft Model

Six-week-old BALB/c nude mice (Beijing HFK Bioscience, Beijing, China) were cared and manipulated according to the agreement approved by Shengjing Hospital of China Medical University. Infected cells (OV-90 or A2780 cells) in the exponential phase were subcutaneously injected into the armpit. After a week, the tumor diameter was measured every three days and calculated for the tumor volume. Mice were sacrificed on the 23^rd^ after being injected. And the tumors were further used for immunohistochemistry staining.

### Immunohistochemistry

Paraffin-embedded sections of tumors were dewaxed in xylene and then rehydrated in gradient alcohol. Then, sections were further maintained in 3% hydrogen peroxide for 15 min to quench the activity of endogenous peroxidase. Sections were incubated with goat serum (Solarbio) for a quarter of an hour at room temperature and then incubated with primary antibodies at 4°C overnight. After rinsed by PBS for three times, these sections were incubated with HRP labeled IgG antibodies (Thermo Scientific, Pittsburgh, PA, USA; 1:500) at 37°C for an hour. Followed by DAB color reaction (Solarbio), sections were stained with hematoxylin (Solarbio) and imaged by microscope (Olympus, Tokyo, Japan). The primary antibodies were ACSM3 (Proteintech; 1:50) and Integrin β1 (Proteintech; 1:50).

### Statistical Analysis

All statistics were analyzed by using GraphPad Prism 8.0 (GraphPad Software, San Diego, CA, USA). Data were presented as means and standard deviation from at least triple independent experiments. One-way ANOVA was used to evaluate statistical differences in multiple groups except for the results of CCK-8 assay and tumor volume (two-way ANOVA). p<0.05 was considered statistically significant.

## Results

### The Expression of *ACSM3* and Its Co-relation of the Overall Survival of Ovarian Cancer

**Figure 1:**
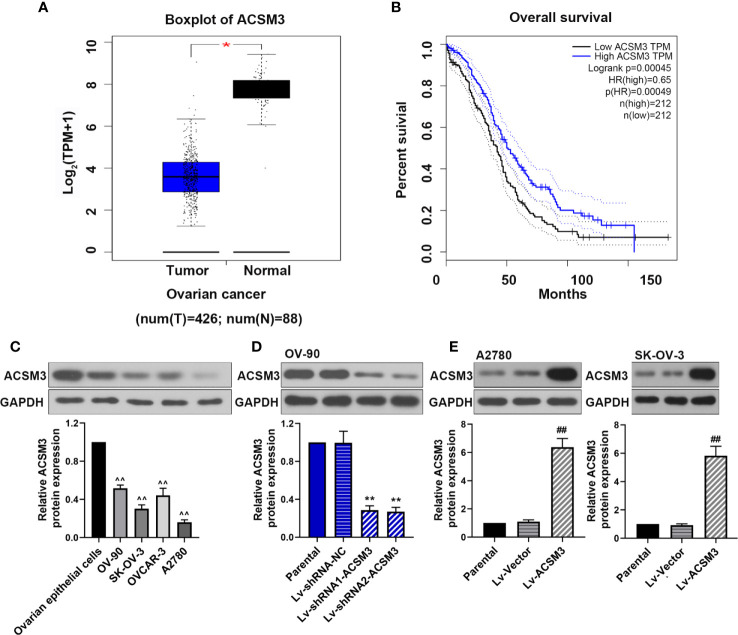
The expression of *ACSM3* and its co-relation of the overall survival of ovarian cancer. **(A)** Boxplot of *ACSM3* in ovarian cancer. TPM, transcripts per kilobase of exon model per million mapped reads. **(B)** Overall survival time between patients with high and low *ACSM3* expression. Dotted lines indicated the 95% confidence interval. HR, hazard ratio. **(C)** Relative protein expression of ACSM3 in ovarian epithelial cells and ovarian cancer cells: OV-90, SK-OV-3, OVCAR-3, and A2780 cells. **(D)** Relative protein expression of ACSM3 in Lv-shRNA-ACSM3 infected OV-90 cells **(E)** Relative protein expression of ACSM3 in Lv-ACSM3 infected A2780 or SK-OV-3 cells. Measurement data were expressed as mean ± SD of three independent experiments. ^^p < 0.01 versus ovarian epithelial cells; *p < 0.05, **p < 0.01 versus Lv-shRNA-NC; ^##^ *p* < 0.01 versus Lv-Vector.

We used Gene Expression Profiling Interactive Analysis (GEPIA; http://gepia.cancer-pku.cn/) to assess the relationship between *ACSM3* expression level and overall survival in ovarian cancers by using The Cancer Genome Atlas database. The results showed that *ACSM3* was markedly low expressed in ovarian cancer tissues than normal tissues (**Figure 1A**) Survival analysis revealed that the low expression level of *ACSM3* was related with poorer overall survival than high mRNA expression of *ACSM3* in 424 ovarian cancer patients, suggesting the prognostic significance of *ACSM3*. (**Figure 1B**). We determined the protein expression of ACSM3 in ovarian epithelial cells and ovarian cancer cells: OV-90, SK-OV-3, OVCAR-3, and A2780 cells. ACSM3 was highly expressed in OV-90 cells and was low expressed in A2780 and SK-OV-3 cells (**Figure 1C**). Then, we artificially regulated the expression of *ACSM3* in OV-90, A2780, and SK-OV-3 cells. Western bolt demonstrated that the knockdown of *ACSM3* in OV-90 cells and the overexpression of *ACSM3* in A2780 or SK-OV-3 cells were successful (**Figures 1D, E**). It could be seen that the expression of *ACSM3* was downregulated in OV-90 cells with a highly expressed baseline of *ACSM3* and was upregulated in SK-OV-3 and A2780 cells with a reduced baseline expression of *ACSM3* (**Figures 1D, E**).

### Effects of *ACSM3* on the Proliferation of Ovarian Cancer Cells

**Figure 2:**
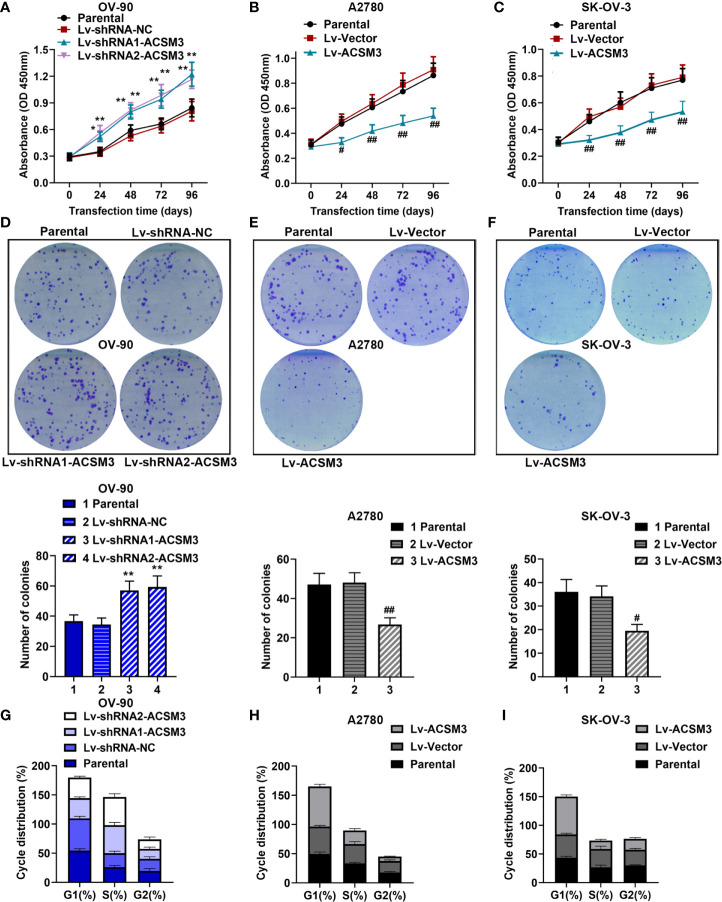
Effects of ACSM3 on the proliferation of ovarian cancer cells. OV-90 cells were infected with *ACSM3* shRNAs. A2780 or SK-OV-3 cells were infected with overexpressed *ACSM3* vectors. **(A–C)** OV-90, A2780, or SK-OV-3 cells were subjected to CCK-8 assay. **(D–F)** The number of colonies. **(G–I)** Cell cycle arrest was detected by the flow cytometer. Measurement data were expressed as mean ± SD of three independent experiments. *p < 0.05, **p < 0.01 versus Lv-shRNA-NC; ^#^P < 0.05, ^##^P < 0.01 versus Lv-Vector.

CCK-8 assay and clone formation assay were performed to measure the ability of cell proliferation in ovarian cancer cells. The results of the CCK-8 assay showed that the knockdown of *ACSM3* promoted the growth of OV-90 cells significantly, and the overexpression of *ACSM3* markedly inhibited the ability of cell proliferation in A2780 or SK-OV-3 cells (**Figures 2A–C**). Clone formation assay demonstrated that the clone forming ability of OV-90 cells was elevated following *ACSM3* knockdown, while the clone forming ability of A2780 or SK-OV-3 cells was decreased by the up-regulation of *ACSM3* (**Figures 2D–F**). To investigate the effects of *ACSM3* on cell cycle arrest, cellular DNA content was detected by flow cytometry in OV-90, A2780, or SK-OV-3 cells. The results showed that the cells in the G1 phase were reduced accompanied by the elevation of cells in the S phase in OV-90 cells induced by the interference of *ACSM3* (**Figure 2G**). And the cells in the G1 phase were enhanced with an attenuation of cells in the S phase and G2 phase in A2780 or SK-OV-3 cells infected with overexpressed *ACSM3* (**Figures 2H, I**). These results indicated *ACSM3* repressed the proliferation ability of ovarian cancer cells.

### Effects of *ACSM3* on Migration and Invasion of Ovarian Cancer Cells

**Figure 3:**
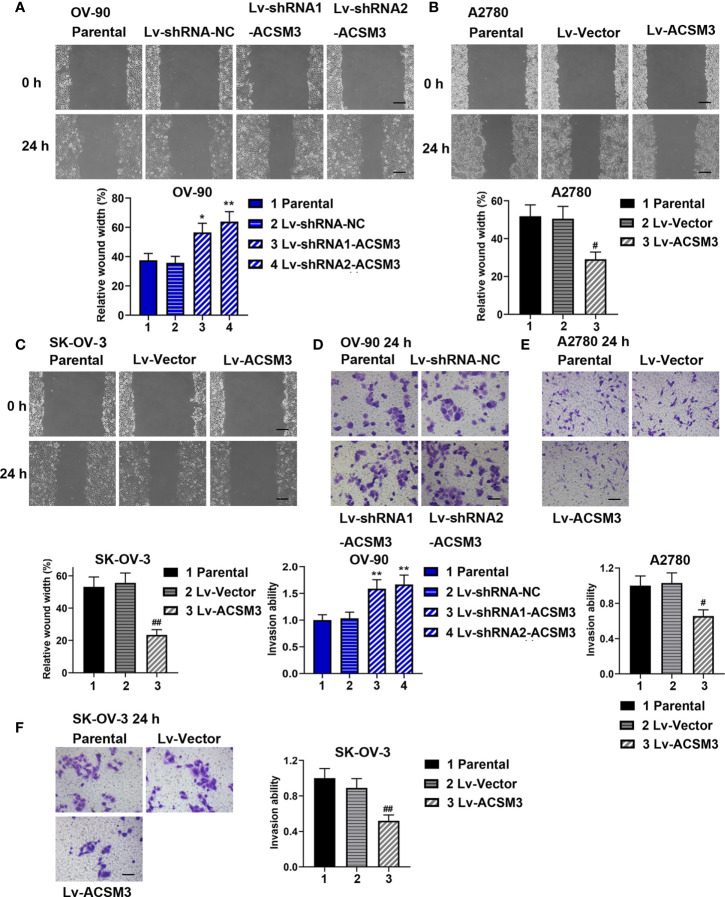
Effects of ACSM3 on migration and invasion of ovarian cancer cells. OV-90 cells were infected with Lv-shRNA-ACSM3. A2780 or SK-OV-3 cells were infected with Lv-ACSM3. **(A-C)** Cell migration ability was measured by wound healing assay. Scale bar represented 200 μm **(D-F)** The ability of cell invasion was detected by transwell assay. Scale bar represented 100 μm. Measurement data were expressed as mean ± SD of three independent experiments. *p < 0.05, **p < 0.01 versus Lv-shRNA-NC; ^#^P < 0.05, ^##^P < 0.01 versus Lv-Vector.

The ability of cell migration and invasion was evaluated by the wound healing assay and the transwell assay. The results of the wound healing assay verified that cell migration ability was enhanced in OV-90 cells induced by the interference of *ACSM3*, while cell migration ability was weakened in A2780 or SK-OV-3 cells infected with Lv-ACSM3 (**Figures 3A-C**). The results of the transwell assay showed that infected with *ACSM3* shRNAs increased the invasion ability of OV-90 cells, while the overexpression of *ACSM3* decreased the invasion ability of A2780 cells or SK-OV-3 cells (**Figures 3D-F**). These data suggested that *ACSM3* decreased the migration and invasion abilities of ovarian cancer cells.

### Effects of *ACSM3* on the Integrin β1/AKT Signaling Pathway in Ovarian Cancer

**Figure 4:**
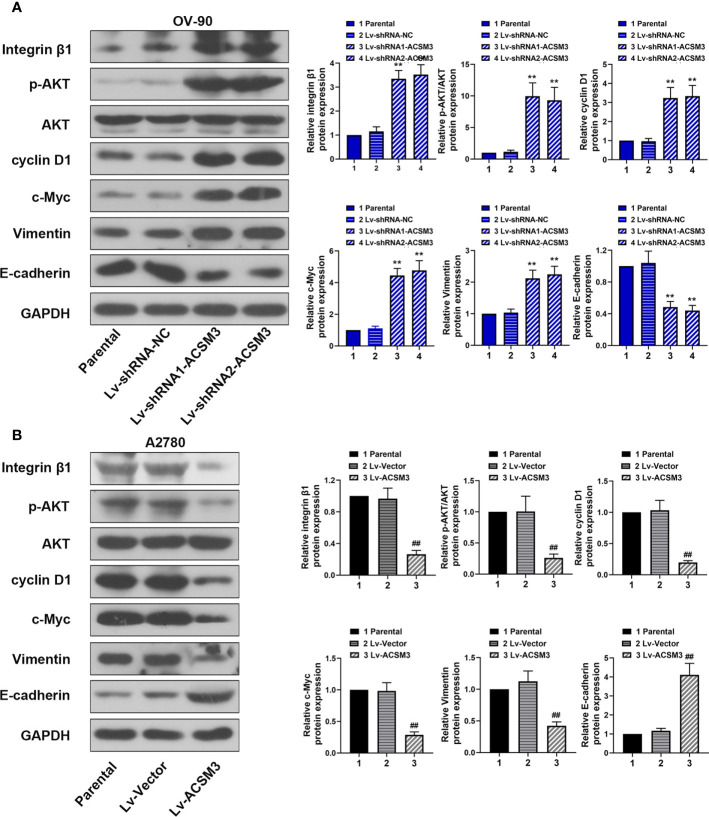
Effects of ACSM3 on the Integrin β1/AKT signaling pathway in ovarian cancer. **(A)** The protein expression of Integrin β1, p-AKT, AKT, cyclin D1, c-Myc, Vimentin, and E-cadherin in OV-90 cells following *ACSM3* knockdown. **(B)** The protein expression of Integrin β1, p-AKT, AKT, cyclin D1, c-Myc, Vimentin, and E-cadherin in A2780 cells following *ACSM3* overexpression. Measurement data were expressed as mean ± SD of three independent experiments. **P < 0.01 versus Lv-shRNA-NC; ^##^P < 0.01 versus Lv-Vector.

To further explore the mechanism of the effects caused by *ACSM3*, the expression of key proteins (Integrin β1, p-AKT, and AKT) in the Integrin β1/AKT signaling pathway was evaluated. Western blot demonstrated that the knockdown of *ACSM3* promoted the expression of Integrin β1 and p-AKT in OV-90 cells, leading to the up-regulation of cyclin D1, c-Myc, Vimentin, and the down-regulation of E-cadherin (**Figure 4A**). The overexpression of *ACSM3* attenuated the expression of Integrin β1 and p-AKT in A2780 cells, causing the low expression of cyclin D1, c-Myc, Vimentin, and the high expression of E-cadherin (**Figure 4B**). Our results suggested the possibility that *ACSM3* attenuated the expression of cell proliferation or metastasis-related proteins by the inhibition of the Integrin β1/AKT signaling pathway in ovarian cancer.

### Effects of *ITG β1* (Integrin β1) on *ACSM3* Up-regulated Ovarian Cancer Cells

**Figure 5:**
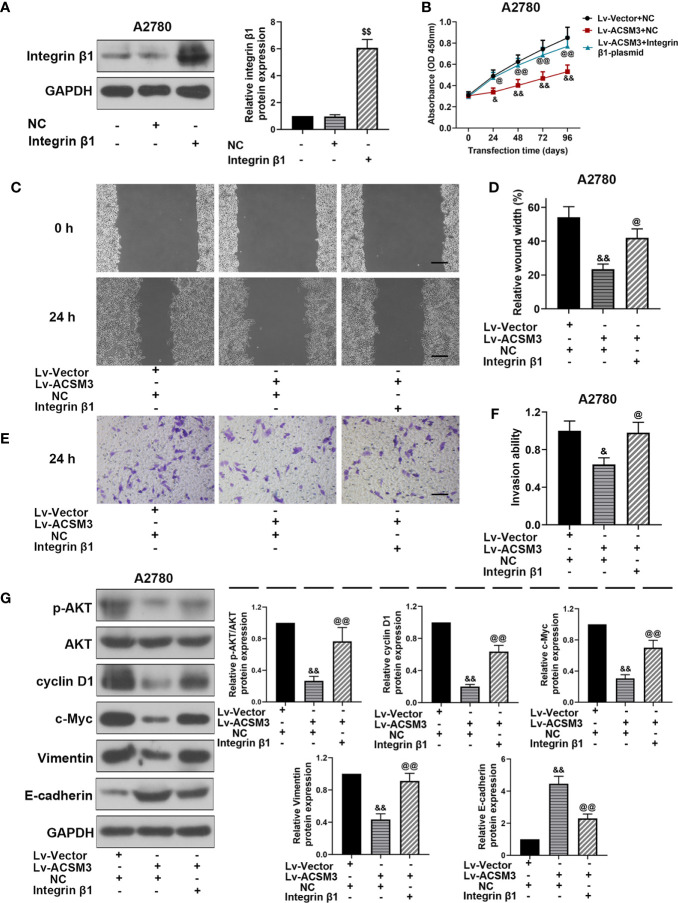
Effects of overexpressed *ITG β1* (Integrin β1) on ACSM3 up-regulated ovarian cancer cells. **(A)** The protein expression of Integrin β1. **(B)** *ACSM3* up-regulated A2780 cells were transfected by *ITG β1* (Integrin β1) overexpression vector and were subjected to CCK-8 assay. **(C, D)** Relative wound width (Scale bar represented 200 μm). **(E, F)** Invasion ability. Scale bar represented 100 μm. **(G)** Relative protein expression of p-AKT, AKT, cyclin D1, c-Myc, Vimentin, and E-cadherin. Measurement data were expressed as mean ± SD of three independent experiments. ^$$^p < 0.01 versus NC; ^&^p < 0.05, ^&&^p < 0.01 versus Lv-Vector+NC; ^@^p < 0.05, ^@@^p < 0.01 versus Lv-ACSM+NC.

To further investigate the effects of Integrin β1 on *ACSM3* up-regulated ovarian cancer cells, *ITG β1* (Integrin β1) overexpressed plasmids were constructed and transfected to A2780 cells. Western blot proved that the construction and transfection of overexpressed plasmids were successful in A2780 cells (**Figure 5A**). The results of the CCK-8 assay revealed that the up-regulated expression of *ACSM3* reduced the growth of A2780 cells. Conversely, the overexpression of *ITG β1* (Integrin β1) reversed the alleviation of cell proliferation ability (**Figure 5B**). *ACSM3* overexpression-induced suppression of cell migration activity was abolished by the overexpression of *ITG β1* (Integrin β1) (**Figures 5C, D**). Moreover, the overexpression of *ITG β1* (Integrin β1) reversed the alleviated cell invasion ability in A2780 cells infected with Lv-ACSM3 (**Figures 5E, F**). Then, we evaluated the effects of overexpressed *ITG β1* (Integrin β1) on the Integrin β1/AKT signaling pathway. *ACSM3* overexpression-induced low expression of p-AKT was reversed by the overexpression of *ITG β1* (Integrin β1), causing the up-regulation of the low expression of cyclin D1, c-Myc, Vimentin and the down-regulation of high expression of E-cadherin (**Figure 5G**). These data confirmed that the overexpressed *ITG β1* (Integrin β1) could reverse the malignant process caused by the up-regulated *ACSM3* in ovarian cancer.

### Effects of *ACSM3* on the Growth of Ovarian Cancer Tumor *In Vivo*

**Figure 6:**
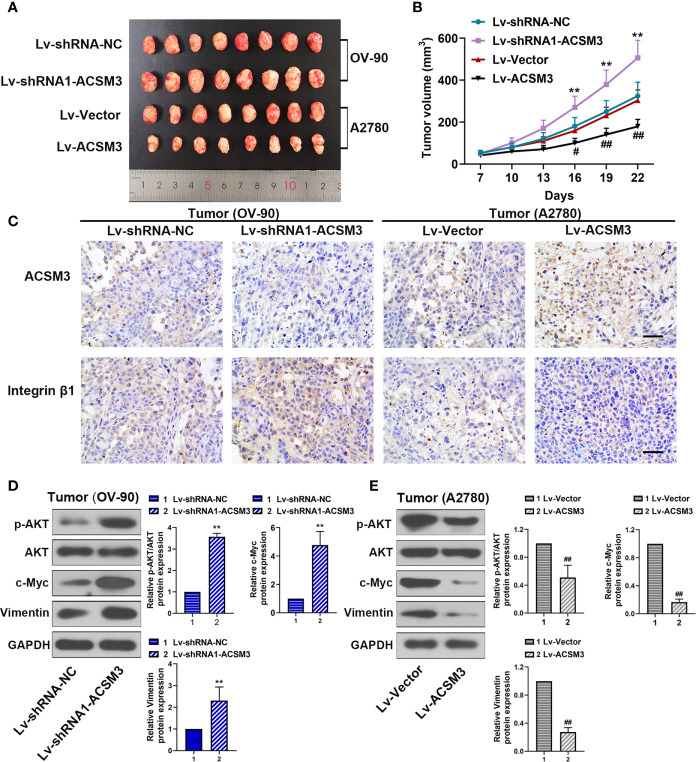
Effects of ACSM3 on the growth of ovarian cancer tumor *in vivo*. A xenograft tumor model was built with OV-90 cells infected with Lv-shRNA1-ACSM3 or A2780 cells infected with Lv-ACSM3. **(A, B)** The volume of xenograft tumors. **(C)** The expression of ACSM3 and Integrin β1 was measured by immunohistochemistry. Scale bar represented 50 μm. **(D, E)** Relative protein expression of p-AKT, AKT, c-Myc, Vimentin, and E-cadherin. Measurement data were expressed as mean ± SD of six independent experiments. **p < 0.01 versus Lv-shRNA-NC; ^#^p < 0.05, ^##^p < 0.01 versus Lv-Vector.

To confirm the effects of *ACSM3* on the growth of ovarian cancer tumor *in vivo*, a xenograft tumor model was built with OV-90 cells infected with Lv-shRNA1-ACSM3 or A2780 cells infected with Lv-ACSM3. Compared to Lv-Vector, the overexpression of *ACSM3* significantly ameliorated the average tumor volume (**Figures 6A, B**). However, compared to Lv-shRNA-NC, *ACSM3* interference markedly increased the average tumor volume (**Figures 6A, B**). Then, we verified the expression of ACSM3 and Integrin β1 by performing immunohistochemistry. The results indicated that the expression of ACSM3 was reduced, while the expression of Integrin β1 was enhanced in tumors derived from the Lv-shRNA1-ACSM3-infected OV-90 cells model (**Figure 6C**). The expression of ACSM3 was increased, while the expression of Integrin β1 was alleviated in tumors derived from the Lv-Vectors-infected A2780 cells model (**Figure 6C**). We further proved the association between ACSM3 and Integrin β1/AKT signaling pathway. The protein expression of p-AKT was elevated in tumors derived from the Lv-shRNA1-ACSM3-infected OV-90 cells model, causing the up-regulation of c-Myc and Vimentin (**Figure 6D**). The protein expression of p-AKT was weakened in tumors derived from the Lv-Vectors-infected A2780 cells model, leading to the attenuated expression of c-Myc and Vimentin (**Figure 6E**). The results suggested that *ACSM3* inhibited the growth of ovarian cancer tumors *in vivo via* the abrogation of the Integrin β1/AKT signaling axis.

## Discussion

In this study, we found that the expression of *ACSM3* was down-regulated in ovarian cancer by bioinformatics analysis, and the patients with low expression of *ACSM3* showed poor overall survival. We artificially regulated the expression of *ACSM3* to evaluate the effects on ovarian cancer progression. Our data revealed that the overexpression of *ACSM3* inhibited cell proliferation, migration, and invasion in ovarian cancer mediated by the suppression of the Integrin β1/AKT signaling pathway *in vitro*. While the knockdown of *ACSM3* enhanced the ovarian cancer progression *via* the activity of the Integrin β1/AKT signaling axis. Moreover, *ACSM3* overexpression-induced suppression of cell migration and invasion activities were abolished by the overexpression of *ITG β1* (Integrin β1) *in vitro.* Then, we found that the overexpression of *ACSM3* ameliorated the growth of ovarian cancer xenograft tumors *in vivo*. Conversely, *ACSM3* interference facilitated the growth of tumors in ovarian cancer.

Studies have shown that the dysregulated expression of acyl-CoA synthetases family subunits exists in many diseases. Acyl-CoA synthetase long-chain family member 4 (ACSL4) is overexpressed in breast and prostate cancer (13). Very long-chain Acyl-CoA synthetase homology 3 (ACSVL3) is seen as a biomarker for targeted therapy in lung cancer (14). Gopal et al (15) found that *ACSM3* was low expressed in hepatocellular carcinoma and activated the WNT/AKT signaling axis. *ACSM3* was reported to have a down-regulated expression in cutaneous melanoma and Duchenne muscular dystrophy (8, 16). However, there have been no studies on the expression of *ACSM3* in ovarian cancer. In this work, we found that the expression of *ACSM3* was down-regulated in ovarian cancer tissues than normal tissues. Furthermore, *ACSM3* has been reported to co-related with poor prognosis in malignant melanoma and liver cancer (7, 8). The survival analysis from the public database showed that the low level of ACSM3 is significantly correlated with the poor overall survival of ovarian cancer, thus, we have reasons to speculate that the ACSM3 is related to the clinic malignancy grade of ovarian cancer. We will attach much importance to this part of the research, and further investigation will be conducted.

In high-grade serous ovarian carcinomas, tumors proliferate rapidly and disseminate early, as well as have an aggressive duration of disease (17). Thus, the inhibition of metastasis is the point for alleviating the disease course of ovarian cancer and for further treatment. Ruan et al (7) reported that the loss of *ACSM3* elevated the metastasis in liver cancer. Zhu et al (8) have proven that the *ACSM3* overexpression decreased the proliferation, invasion, and colony formation in malignant melanoma *in vitro*. In this research, we artificially regulated the expression of *ACSM3* to determine the effects it on the progression of ovarian cancer *in vitro* or *in vivo*. The results revealed that the overexpression of *ACSM3* significantly decreased the abilities of ovarian cell proliferation, migration, and invasion *in vitro*, and weakened the growth of ovarian cancer tumors *in vivo*.

Furthermore, we determined the mechanism that *ACSM3* acted as an antioncogene in ovarian cancer. The up-regulated expression of *ITG β1* (Integrin β1) has been proven to be linked with ovarian cancer progressions such as cell proliferation, metastasis, and invasion (18, 19). AKT is known as a vital part of the cell cycle, cell survival, and apoptosis, and is positively associated with Integrin β1 (20). Studies revealed that the high expression of E-cadherin and the low expression of Vimentin cause epithelial differentiation, which is essential for tumor metastasis and invasion (21). The gain of cyclin D1 and c-Myc are associated with cell proliferation and malignant transformation (22). Zhang et al (23) reported that the loss of the Integrin β1/FAK/AKT signaling axis caused the down-regulation of cyclin D1 and collagen in hepatic fibrosis rats. Riggio et al. (24) pointed out that the AKT1 enhanced cell proliferation *via* the up-regulated cyclin D1 and *S6*, and then suppressed the cell migration and invasion *via* the down-regulated Integrin β1 and FAK. Bartolomé et al. (25) demonstrated that integrin signal specifically activated AKT and JNK mediated by vascular-endothelial (VE)-cadherin, which was associated with metastatic dissemination in melanoma. Here, we found that the overexpressed *ACSM3* blocked the expression of Integrin β1 and p-AKT, leading to the down-regulation of cyclin D1, c-Myc, Vimentin and the up-regulation of E-cadherin in ovarian cancer cells. These results indicated that up-regulated *ACSM3* inhibited the cell proliferation, migration, and invasion *via* the Integrin β1/AKT signaling pathway in ovarian cancer. Moreover, the *ACSM3* overexpression-induced suppression of cell migration and invasion activities were abolished by the overexpression of *ITG β1* (Integrin β1).

## Conclusion

*ACSM3* was low expressed in ovarian cancer tissues. The overexpression of *ACSM3* inhibits the malignant phenotypes *via* the Integrin β1/AKT signaling axis in ovarian cancer. Furthermore, *ACSM3* could be a potential therapeutic target in ovarian cancer.

## Data Availability Statement

The raw data supporting the conclusions of this article will be made available by the authors, without undue reservation.

## Ethics Statement

The animal study was reviewed and approved by the Ethics Committee of China Medical University.

## Author Contributions

LY performed the experiments and wrote the manuscript. ZH and WL performed the statistical analysis. NL wrote sections of the manuscript. SG designed the experiment and made revision of the manuscript. All authors contributed to the article and approved the submitted version.

## Funding

This study was supported by grants from the 345 Talent Project, Shengjing Hospital of China Medical University and Young Backbone Teachers Projects of China Medical University QGZD2018063, 345 Talent Project.

## Conflict of Interest

The authors declare that the research was conducted in the absence of any commercial or financial relationships that could be construed as a potential conflict of interest.
